# Ragweed Subpollen Particles of Respirable Size Activate Human Dendritic Cells

**DOI:** 10.1371/journal.pone.0052085

**Published:** 2012-12-14

**Authors:** Kitti Pazmandi, Brahma V. Kumar, Krisztina Szabo, Istvan Boldogh, Arpad Szoor, Gyorgy Vereb, Agota Veres, Arpad Lanyi, Eva Rajnavolgyi, Attila Bacsi

**Affiliations:** 1 Department of Immunology, Medical and Health Science Center, University of Debrecen, Debrecen, Hungary; 2 Department of Microbiology and Immunology, University of Texas Medical Branch at Galveston, Galveston, Texas, United States of America; 3 Department of Biophysics and Cell Biology, Medical and Health Science Center, University of Debrecen, Debrecen, Hungary; Cardiff University School of Medicine, United Kingdom

## Abstract

Ragweed (*Ambrosia artemisiifolia*) pollen grains, which are generally considered too large to reach the lower respiratory tract, release subpollen particles (SPPs) of respirable size upon hydration. These SPPs contain allergenic proteins and functional NAD(P)H oxidases. In this study, we examined whether exposure to SPPs initiates the activation of human monocyte-derived dendritic cells (moDCs). We found that treatment with freshly isolated ragweed SPPs increased the intracellular levels of reactive oxygen species (ROS) in moDCs. Phagocytosis of SPPs by moDCs, as demonstrated by confocal laser-scanning microscopy, led to an up-regulation of the cell surface expression of CD40, CD80, CD86, and HLA-DQ and an increase in the production of IL-6, TNF-α, IL-8, and IL-10. Furthermore, SPP-treated moDCs had an increased capacity to stimulate the proliferation of naïve T cells. Co-culture of SPP-treated moDCs with allogeneic CD3^+^ pan-T cells resulted in increased secretion of IFN-γ and IL-17 by T cells of both allergic and non-allergic subjects, but induced the production of IL-4 exclusively from the T cells of allergic individuals. Addition of exogenous NADPH further increased, while heat-inactivation or pre-treatment with diphenyleneiodonium (DPI), an inhibitor of NADPH oxidases, strongly diminished, the ability of SPPs to induce phenotypic and functional changes in moDCs, indicating that these processes were mediated, at least partly, by the intrinsic NAD(P)H oxidase activity of SPPs. Collectively, our data suggest that inhaled ragweed SPPs are fully capable of activating dendritic cells (DCs) in the airways and SPPs' NAD(P)H oxidase activity is involved in initiation of adaptive immune responses against innocuous pollen proteins.

## Introduction

Allergic asthma, a chronic inflammatory disorder of the airways, affects over 300 million individuals worldwide and an estimated 35 million in the United States alone [1]. One of the most common triggers of allergic asthma is the pollen of short ragweed (*Ambrosia artemisiifolia*). Ragweed is ubiquitous in North America, and, in recent decades, it has spread rapidly across Europe [2]. The prevalence of allergic asthma has increased over the last 40 years [3]; however, the reasons for this phenomenon cannot yet be explained. Possible explanations include the expansion of ragweed species due to increasing deforestation and economic development [4], and elevated pollen production and growing pollen season length associated with climate change [5], [6]. The biochemistry of pollen allergens and the mechanism by which they trigger clinical symptoms in sensitized individuals are relatively well understood; however, several unresolved questions remain relating to the development of adaptive immune responses against pollen-derived proteins.

Although there is unequivocal evidence that pollen antigens can induce allergic inflammation throughout the respiratory tract, whole pollen grains are considered too large to reach the lower airways [7]. Further, there is a poor correlation between measured airborne allergen levels and related pollen counts, indicating that pollen grains may differ in allergen release [8]; moreover, significant allergen exposure can occur even in the absence of identifiable airborne pollen grains [9]. Finally, even though levels of airborne pollen grains decrease following summer thunderstorms, the incidence of allergic reactions in sensitive individuals increases [10]. These contradictory phenomena can be explained by the fact that upon hydration, whole pollen grains release SPPs of respirable size (<5 μm). The release of such SPPs has been reported in short ragweed [11] and several other species [12]–[16].

It has been previously demonstrated that the released SPPs with sizes ranging from 0.5 to 4.5 µm retain key components necessary to induce airway inflammation [11], [17], [18]. Specifically, ragweed SPPs contain Amb a 1, the predominant allergen in ragweed pollen against which 90% of sensitized subjects have antibodies [19]. Ragweed SPPs also possess NAD(P)H oxidases, which catalyze the production of superoxide anions in a manner similar to that of mammalian phagocyte NADPH oxidases [20] and induce profound oxidative stress in the lungs or conjunctiva within minutes after exposure [21], [22]. This oxidative insult and pollen allergens act together to initiate robust allergic inflammation in sensitized subjects [21].

DCs form a network in the lungs and sample inhaled antigens. If DCs sense danger signals during antigen uptake, they become activated and release pro-inflammatory cytokines and chemokines that initiate rapid innate immune responses. Activated DCs down-regulate molecules involved in antigen capture, up-regulate major histocompatibility complex molecules, start to express co-stimulatory molecules, and migrate to the draining lymph nodes to encounter and activate naïve T lymphocytes [23]. Thus, the initiation of allergic sensitization depends mainly on the ability of inhaled allergens to generate danger signals that activate DCs. Recently we found that ROS produced by intact pollen grains are able to launch the activation program of human monocyte-derived DCs [24]; however, the involvement of inhaled pollen grains in the sensitization phase of allergic reactions *in vivo* is still far from being fully explored.

Here we demonstrate that ragweed SPPs, which easily penetrate deep into the lungs, can be captured by DCs. Furthermore, SPPs' NAD(P)H oxidase activity elevates intracellular ROS levels upon exposure leading to the activation of DCs. Oxidative stress-activated DCs are able to trigger naïve T cell proliferation and cytokine release from effector T cells. Thus, NAD(P)H oxidase activity of SPPs may possess pivotal role in development of adaptive immune responses against innocuous pollen proteins.

## Materials and Methods

### Ethics statement

Peripheral blood mononuclear cells (PBMCs) were isolated from heparinized leukocyte-enriched buffy coats of healthy blood donors drawn at the Regional Blood Center of Hungarian National Blood Transfusion Service (Debrecen, Hungary), with the written approval of the Director of the National Blood Transfusion Service and the Regional and Institutional Ethics Committee of the University of Debrecen, Medical and Health Science Center (Debrecen, Hungary). Written informed consent was obtained from donors prior to blood donation, and their data were processed and stored according to the principles expressed in the Declaration of Helsinki.

### Cells

Human PBMCs were isolated by centrifugation on a Ficoll-Paque (GE Healthcare, Uppsala, Sweden) density gradient, and monocytes were separated from PBMCs using magnetic anti-CD14 microbeads (Miltenyi Biotec, Bergisch-Gladbach, Germany), according to the manufacturer's instructions. Purity of the monocyte fraction was ≥97%, as determined by flow cytometric analysis.

Human CD3^+^ pan-T cells were isolated from PBMCs using magnetic anti-CD3 microbeads (Miltenyi Biotec), according to the manufacturer's instructions. Purity of the CD3^+^ T cell fraction was ascertained by flow cytometry to be ≥98%.

Untouched human naïve CD4^+^CD45RA^+^CD45RO^-^ T cells were separated from PBMCs with a naïve CD4^+^ T Cell Isolation Kit II (Miltenyi Biotec), according to the manufacturer's instructions. This kit is specifically designed to leave naïve CD4^+^ T cells untouched by removing CD4^+^CD45RO^+^ memory T cells, non-CD4^+^ T cells, and all other cell types present in the PBMC fraction. The final purity of untouched naïve CD4^+^ T cells was ≥98%, as assessed by flow cytometry.

### Generation of moDC

Freshly isolated monocytes were cultured in 24-well tissue culture plates at a density of 2×10^6^ cells/ml in RPMI (Sigma-Aldrich, St. Louis, MO) supplemented with 2 mM L-glutamine (Sigma-Aldrich), 100 U/ml penicillin, 100 ng/ml streptomycin, and 10% heat-inactivated FCS (Invitrogen, Carlsbad, CA). Cells were stimulated with 80 ng/ml GM-CSF (Gentaur Molecular Products, Brussels, Belgium) and 100 ng/ml IL-4 (Peprotech EC, London, UK) immediately and on day 2. Cells were used for experiments on day 5, at which point ≥90% expressed immature DC phenotype (DC-SIGN/CD209^+^, CD14^low^) and ≥80% were CD1a positive.

### Isolation of SPPs

Short ragweed (*Ambrosia artemisiifolia*) pollen was obtained from Greer Laboratories (Lenoir, NC) and subpollen particles were isolated as previously described [11]. Briefly, whole pollen grains were hydrated in sterile, pyrogen-free deionized water, and the resulting suspension was vortexed and rotated for 90 min at room temperature. Intact pollen grains and pollen fragments were removed by centrifugation (1600 g, 5 min, 4°C). The supernatant was filtered (5-μm filter, Sartorius Stedim Biotech, Göttingen, Germany) and centrifuged (9000 g, 15 min, 4°C). The resulting pellets were collected and resuspended in sterile PBS (PAA Laboratories GmbH, Pasching, Austria). To determine the number of SPPs, an improved Neubauer chamber was used, as previously described [25]. PyroGene^TM^ Endotoxin Detection Assay Kit (Lonza Group Ltd., Basel, Switzerland) was utilized to test the endotoxin level of the SPP suspension. After one-step centrifugation the supernatant of the SPP suspension was collected and its protein concentration was determined using a Pierce BCA Protein Assay Kit (Thermo Fisher Scientific, Rockford, IL).

### Measurement of ROS generated by SPPs

2′–7′-dihydro-dichlorofluorescein diacetate (H_2_DCF-DA, Molecular Probes, Eugene, OR) was used to detect ROS production by SPPs. Samples containing freshly isolated SPPs and 50 μM H_2_DCF-DA working solution were incubated in 96-well plates for 1 h at 37°C. Fluorescence intensity measurements were taken using a Synergy HT micro plate reader (Bio-Tek Instruments, Winooski, VT) at 485 nm excitation and 528 nm emission. To prove that ROS generation was due to NAD(P)H oxidases present in SPPs, control experiments were carried out with heat-inactivated (72°C, 30 min) SPPs (SPP^H^) and the supernatant of the freshly isolated SPPs (SPP^SUP^) with or without 100 µM NADPH, a substrate of the enzymes, or 5 µM DPI, an NADPH oxidase inhibitor (both from Sigma-Aldrich).

### Assessment of intracellular ROS levels

Human moDCs were incubated with freshly isolated SPPs at a ratio of 1∶15 (moDC/SPP) for 1 h at 37°C, both in the presence and absence of 100 µM NADPH. An optimum ratio was determined in preliminary experiments. In control experiments cells were exposed to SPP^H^ or SPPs treated with DPI (5 µM; Sigma-Aldrich). SPPs were exposed to DPI for 30 min, and then excess DPI was removed by centrifugation. After incubation, cells were washed twice with PBS and then loaded with H_2_DCF-DA (Molecular Probes) for 25 min at 37°C. Fluorescence intensities were assessed with a FACS Calibur Flow Cytometer (Becton Dickinson, Franklin Lakes, NJ) and data analysis was performed using FlowJo Software (Treestar, Ashland, OR).

### SPP uptake by human moDCs

SPPs were fluorescently labeled with CellVue® Jade Dye (45 min, 4°C; Polysciences Inc., Warrington, PA). To eliminate excess probe, SPPs were transferred to a separate vial, washed twice, pelleted by centrifugation (9000 g, 15 min, 4°C), and resuspended in PBS.

Human moDCs were seeded in 24-well tissue culture plates at a density of 10^6^ cells/ml, and incubated for 4 h with fluorescently-labeled SPPs at either 4°C or 37°C. The percentage of CellVue® Jade positive cells was determined by flow cytometry, with a minimum of 10,000 cell counts per sample. The fluorescence of untreated cells was used as a threshold level above which cells were considered to have internalized/attached SPPs.

Confocal laser scanning microscopy (Zeiss LSM 510 microscope, Carl Zeiss AG, Jena, Germany and Zeiss AIM 4.2 software, Carl Zeiss Microimaging) was used to determine the cellular localization of SPPs in treated moDCs. After treatment with fluorescently-labeled SPPs, PE-conjugated anti-hDC-SIGN (R&D System, Minneapolis, MN)-labeled and formaldehyde-fixed cells were mounted on microscopic slides with Mowiol 4–88 (Calbiochem, Darmstadt, Germany) under the coverslip to reduce unwanted photobleaching. CellVue® Jade-labeled SPPs were excited at 488 nm, and PE-conjugated anti-hDC-SIGN stained cells were excited at 543 nm. Fluorescence emission was detected through 505 to 550 nm and 560 to 615 nm band-pass filters. Images were taken in multi track mode to prevent cross-talk. Image stacks of 512×512-pixel, 1.5-μm thick optical sections were obtained with a 40× C-Apochromat water immersion objective (NA1.2).

### Phenotype of human moDCs

To evaluate cell surface protein expression of moDCs, the following antibodies were used: anti-CD40-FITC (BD Pharmingen, San Diego, CA), anti-CD80-FITC, anti-HLA-DQ-PE (both from BioLegend, Uithoorn, The Netherlands), anti-CD86-PE (R&D System, Minneapolis, MN), and isotype-matched control antibodies (BD Pharmingen). Fluorescence intensities were measured with a FACS Calibur Flow Cytometer (Becton Dickinson).

### Cytokine and chemokine measurements

Cytokine and chemokine release from moDCs was determined by ELISA at 24 h after treatments. Assay kits specific for IL-1β, IL-6, IL-8, IL-10, IL-12(70) and TNF-α (all from BD Biosciences, San Diego, CA) were used according to the manufacturer's instructions. IL-10 and TGF-β1 ELISA kits (both from BD Biosciences) were utilized to measure the cytokine levels in the CD3^+^ pan-T cells/moDC co-cultures' supernatants collected on day 4. Absorbance measurements were obtained with a Synergy HT micro plate reader (Bio-Tek Instruments) at 450 nm.

### Proliferation of naïve CD4^+^ T cells

Immature moDCs (2×10^5^ cells/well in 96-well tissue culture plates) were pre-incubated with SPPs, DPI-treated SPPs, and SPP^H^ for 24 h in the presence and absence of NADPH. After pre-incubation, moDCs were washed twice with cell culture medium and then co-cultured with freshly isolated, allogeneic naïve CD4^+^ T cells, which were previously labeled with 0.5 µM CFSE (Invitrogen), for 5 days in the presence of 1 µg/ml purified anti-human CD3 mAb (Clone: HIT3a, BD Pharmingen) at a ratio of 1∶20 (moDC-T cell). After co-cultivation, fluorescence intensities were detected on the FL1 (530±15 nm) channel of a BD FACSCalibur flow cytometer (Becton Dickinson) and data were analyzed by FlowJo software (Treestar).

### Cytokine secretion of CD3^+^ pan-T cells

Freshly isolated CD3^+^ pan-T cells from three ragweed allergic individuals and three non-allergic ones were co-cultured with allogeneic moDCs at a ratio of 1∶10 (moDC/T cell) in 48-well tissue culture plates for 4 days. Before co-culturing, the allogeneic moDCs were pre-incubated with intact and DPI-treated SPPs in the presence and absence of NADPH for 24 h and then washed twice with cell culture medium. Ragweed allergic and non-allergic donors were selected after screening for total and ragweed protein-specific IgE levels outside of the pollen season. Non-allergic donors were characterized by low levels of total IgE (<20 IU/ml) and ragweed-specific IgE (<0.35 kU/l). Donors with elevated ragweed-specific IgE levels (>0.70 kU/l) and a positive history of seasonal allergic rhinitis were classified as “ragweed allergic” individuals. To determine the percentage of T cells secreting cytokines in response to the moDC priming, ELISPOT assay was performed using human IFN-γ, IL-17, and IL-4 ELISPOT kits (all from eBioscience, San Diego, CA) as previously described [26]. The formation of colored spots was detected with an ImmunoScan analyzer using ImmunoSpot 4.0 software (C.T.L.-Cellular Technology Ltd., Bonn, Germany).

### Statistical analysis

Data were analyzed by Student's paired *t* test or ANOVA, followed by Bonferroni *post hoc* test. Data analysis was performed with SPSS version 12.0 for Windows (SPSS Inc., Chicago, IL). Differences were considered to be statistically significant at *p*<0.05.

## Results

### SPPs' ROS production is due to their intrinsic NAD(P)H oxidase activity

**Figure 1:**
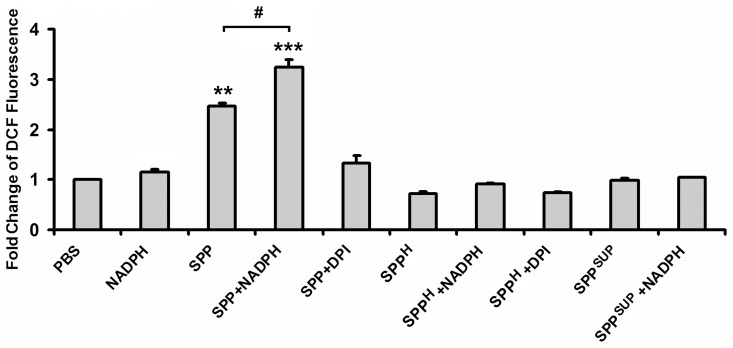
ROS generating capacity of freshly isolated SPPs. To determine ROS production by SPPs, H_2_DCF-DA, a redox-sensitive dye, was added to the SPP suspension and the changes in DCF fluorescence intensity were measured by fluorometry. To prove that ROS production was due to intrinsic NAD(P)H oxidases present in SPPs, controls containing heat-inactivated SPPs, the supernatant of the freshly isolated SPPs, NADPH, a substrate of NAD(P)H oxidases, and DPI, an NADPH oxidase inhibitor, were included. Data are presented as means ± SEM of three individual experiments. ** *p*<0.01; *** *p*<0.001 vs PBS, ^#^ *p*<0.05. (DCF: dichlorofluorescein; DPI: diphenyleneiodonium; SPP: subpollen particle; SPP^H^: heat-inactivated SPP; SPP^SUP^: supernatant of SPPs).

The redox-sensitive H_2_DCF-DA was utilized to test the ability of ragweed SPPs to generate ROS. Intact SPPs induced a 2.5-fold increase in dichlorofluorescein (DCF) fluorescence as compared to PBS control (Fig. 1). Addition of NADPH to SPPs significantly (*p*<0.05) elevated their ROS production. ROS generation by SPPs was significantly (*p*<0.01) decreased in the presence of DPI (an NADPH oxidase inhibitor) and completely eliminated by heat-treatment (Fig. 1). ROS production in the supernatant of SPPs could not be detected either in the presence or absence of NADPH (Fig. 1). These findings indicate that NAD(P)H oxidases in isolated SPPs are responsible for the detected ROS generation.

### Exposure to SPPs induces oxidative stress in human moDC

**Figure 2:**
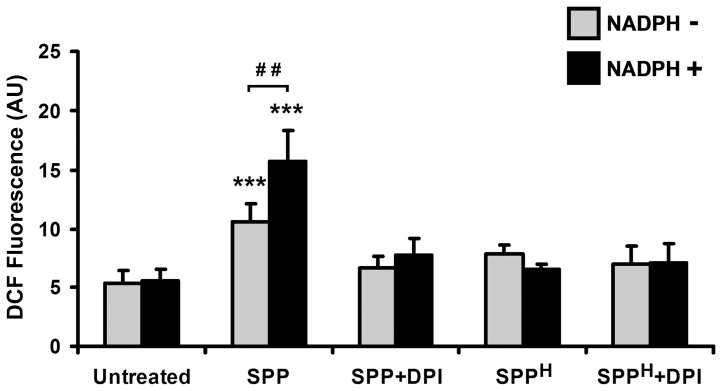
Exposure to SPPs increases the intracellular ROS levels in human moDCs. Cells were incubated with freshly isolated ragweed SPPs for 1 h both in the presence and absence of NADPH. Cells were then loaded with the redox-sensitive H_2_DCF-DA and changes in DCF fluorescence intensities were assessed by flow cytometry. Control experiments were performed using heat-inactivated SPPs and SPPs treated with DPI, an inhibitor of NADPH oxidases. Data are presented as means ± SEM of three individual experiments. *** *p*<0.001 vs untreated DCs, ^##^ *p*<0.01. (AU, arbitrary units; DCF: dichlorofluorescein; DPI: diphenyleneiodonium; SPP: subpollen particle; SPP^H^: heat-inactivated SPP).

In our experiments, *ex vivo*-generated human moDCs, which express CD1a, CD1c (BDCA1), CD11c, and MHCII [27], were used to mimic the function of the myeloid DC type 1 (BDCA1^+^, HLA-DR^+^, CD11c^+^) subset, a strong inducer of T cell proliferation, in human lung [28], [29]. While the majority of these CD1c^+^ DCs also express CD1a, separate small populations of CD1c^+^ CD1a^-^ and CD1c^-^ CD1a^+^ DCs could consistently be identified in the lung [28]. CD1a^+^ DCs were reported to localize within the epithelial layer of the airways, whereas CD1c^+^ DCs were almost exclusively in the submucosa. Cells expressing both CD1a and CD1c were noted at both locations [30]. To test whether SPP treatment affects intracellular ROS levels in human moDCs, cells were incubated with SPPs for 1 h under various conditions and then loaded with H_2_DCF-DA. SPP exposure significantly increased (2.0±0.2-fold, *p*<0.001) intracellular DCF fluorescence (Fig. 2). As SPPs possess NAD(P)H oxidase activity, treatments were also performed in the presence of NADPH. Addition of NADPH further increased (2.9±0.4-fold, *p*<0.01) the ability of SPPs to induce oxidative stress in moDCs, while NADPH itself did not modify intracellular DCF signals (Fig. 2). When cells were exposed to SPPs treated with DPI (a NADPH oxidase inhibitor), SPP^H^, or SPP^H^ treated with DPI, only a minimal increase in DCF fluorescence could be detected, suggesting that the observed increase in the intracellular ROS levels was primarily induced by the NAD(P)H oxidase activity of SPPs.

### Human moDCs internalize SPPs

**Figure 3:**
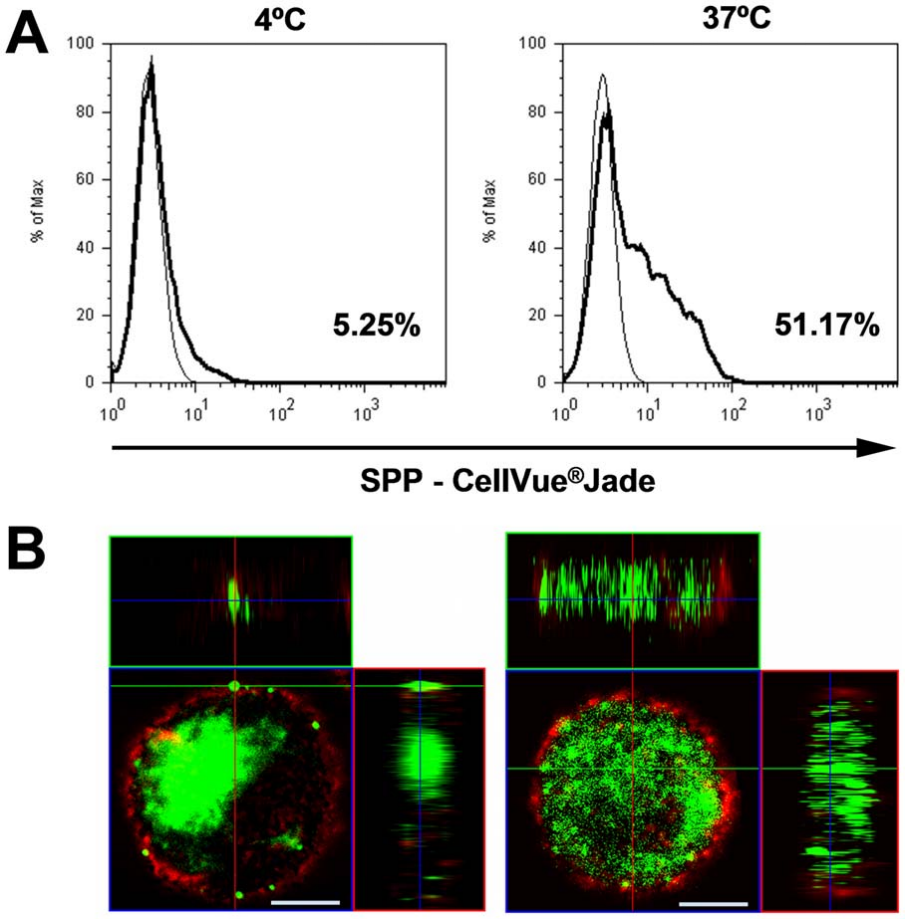
Uptake of SPPs by human moDCs. (A) Investigation of the percentage of human moDCs having attached/internalized SPPs by means of flow cytometry. Cells were incubated for 4 h with CellVue® Jade-labeled SPPs at 37°C or 4°C. The fluorescence of untreated cells (thin line) was used as a threshold level above which cells were considered to have attached SPPs (thick line). Numbers indicate the percentage of SPP attached/internalized cells in a representative measurement from four independent experiments. (B) Visualization of SPPs in human moDCs by confocal laser scanning microscopy. Human moDCs were cultivated with CellVue® Jade-labeled SPPs (green) for 4 h, stained with PE-conjugated anti-hDC-SIGN (red), fixed, and visualized by confocal microscopy. Cells are shown at 40× magnification. Scale bar  = 5 µm.

To investigate whether SPPs are attached to or internalized by moDCs, cells were treated with fluorescently-labeled SPPs for 4 h at either 4°C or 37°C. Depending on the donor, 25–58% of moDCs showed internalization/attachment of SPPs at 37°C (Fig. 3A). Only a small percentage of moDCs were SPP-positive after incubation at 4°C, indicating that SPPs were attached/internalized by active processes. To distinguish between attachment and internalization, confocal laser scanning microscopy was performed. The cross-sectional images of SPP-positive cells revealed that SPPs were either exclusively internalized or simultaneously internalized and attached to the cell surface (Fig. 3B). SPP-positive cells without internalization could not be detected.

### ROS generated by SPPs' NAD(P)H oxidases trigger phenotypic changes in human moDCs

**Figure 4:**
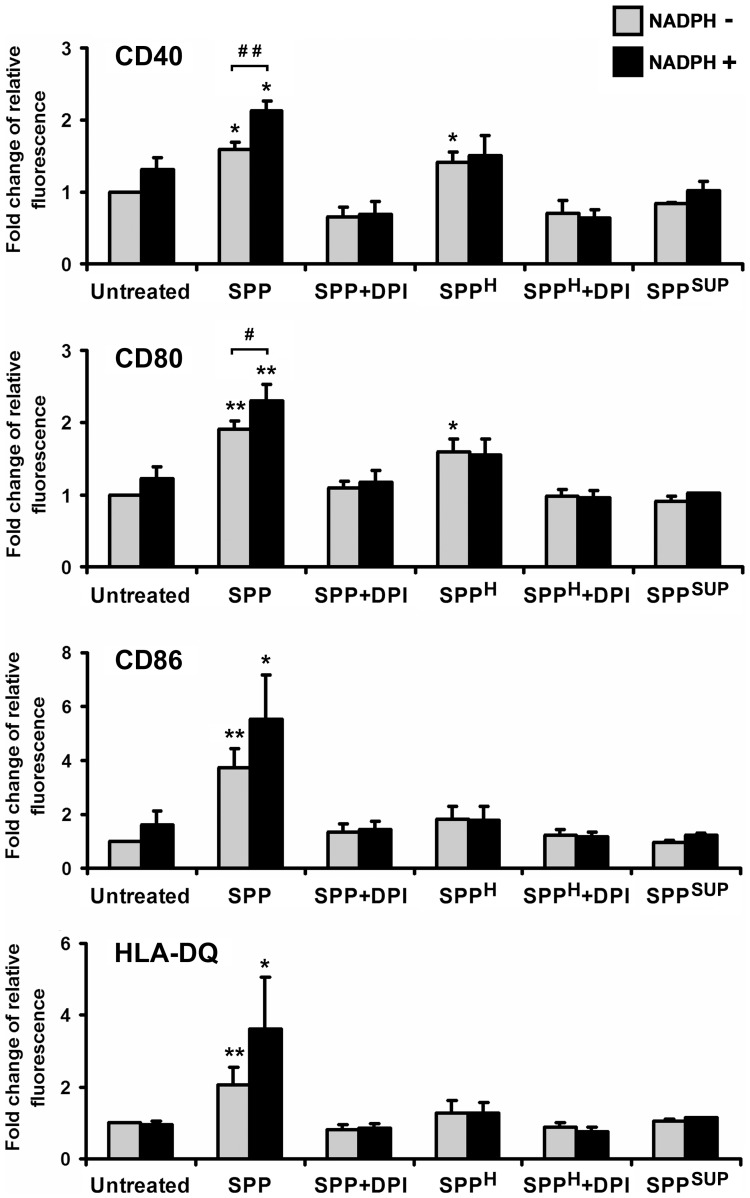
Phenotypic characterization of SPP-exposed human moDCs. Cells were treated with freshly isolated SPPs, DPI-treated SPPs, and heat-inactivated SPPs, individually and in combination with NADPH for 24 h. Expression of HLA-DQ and co-stimulatory molecules was analyzed by means of flow cytometry. Data are presented as means ± SEM of four independent experiments. * *p*<0.05; ** *p*<0.01; *** *p*<0.001 vs untreated DCs, ^#^ *p*<0.05; ^##^ *p*<0.01. (DPI: diphenyleneiodonium; SPP: subpollen particle; SPP^H^: heat-inactivated SPP; SPP^SUP^: supernatant of SPPs).

To evaluate the phenotypic changes of immature moDCs triggered by exposure to SPPs, the expressions of CD40, CD80, CD86 (all co-stimulatory molecules), and HLA-DQ (an antigen-presenting molecule) were measured by flow cytometry. Treatment with SPPs markedly increased the expressions of CD40 (*p*<0.05), CD80 (*p*<0.01), CD86 (*p*<0.01), and HLA-DQ (*p*<0.01) (Fig. 4). When cells were exposed to SPPs in the presence of NADPH, a further increase in the expression of all tested molecules was detected; however, the changes were significant only in cases of CD40 and CD80 (*p*<0.01 and *p*<0.05, respectively). Insignificant phenotypic changes of moDCs were detected when using SPPs treated with DPI, SPP^H^, or SPP^H^ treated with DPI, except effects of SPP^H^ on CD40 and CD80 expression (Fig. 4). In control experiments, exposure to SPPs' supernatant containing 28 pg/ml endotoxin as measured by a commercial assay kit (see Materials and Methods) did not modify the phenotypic characteristics of moDCs (Fig. 4). These findings suggest that the phenotypic changes of SPP-treated moDCs were induced at least partially by SPPs' NAD(P)H oxidase activity.

### Exposure to SPPs upregulates IL-6, TNF-α, IL-8, and IL-10 secretion by human moDCs

**Figure 5:**
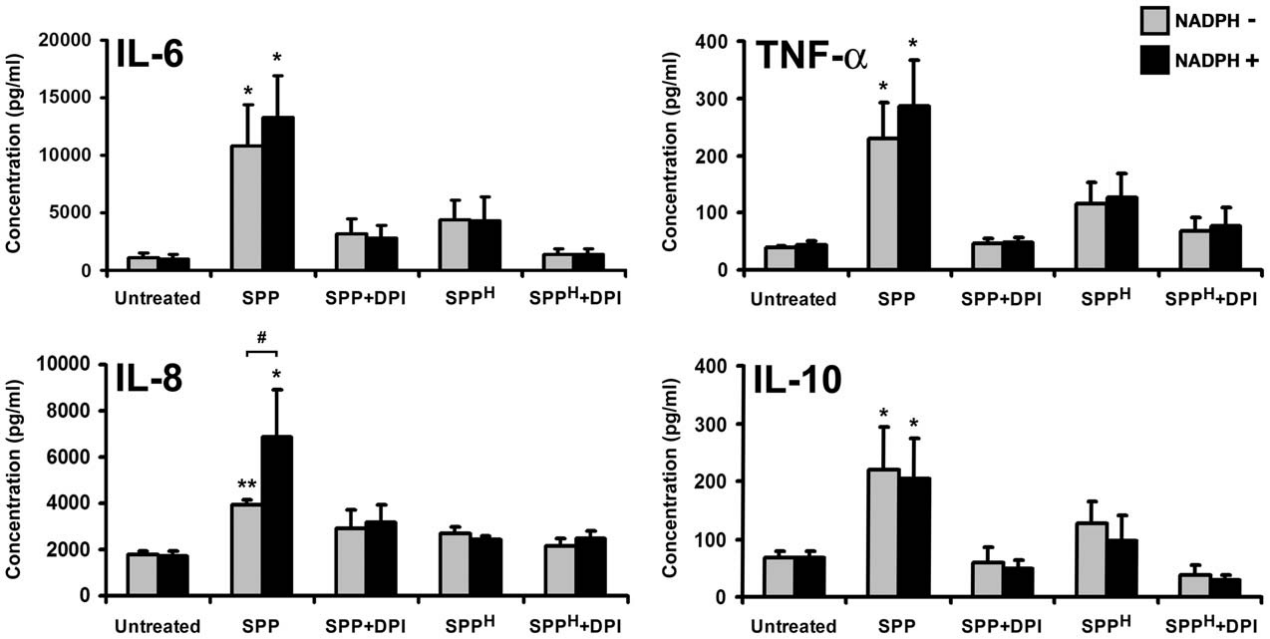
Cytokine and chemokine profile of human moDCs exposed to SPPs. ELISA was used to determine the release of cytokines (IL-6, TNF-α, IL-10) and chemokine (IL-8) from human moDCs in response to freshly isolated SPPs, DPI-treated SPPs, and heat-inactivated SPPs, in the presence and absence of NADPH, after 24 h incubation. Data are presented as means ± SEM of four individual experiments. * *p*<0.05; ** *p*<0.01 vs untreated DCs, ^#^ *p*<0.05. (DPI: diphenyleneiodonium; SPP: subpollen particle; SPP^H^: heat-inactivated SPP).

Next, we examined cytokine and chemokine production by moDCs exposed to SPPs. Secretion of IL-1β, IL-6, TNF-α, IL-12(70), IL-8, and IL-10 was measured by ELISA. Administration of SPPs or SPP^H^ did not significantly modify the very low basal levels of IL-1β and IL-12 released by moDCs (data not shown). Compared with untreated controls, moDCs treated with SPPs secreted significantly higher levels of IL-6 (11.1±4.2-fold increase, *p*<0.05), TNF-α (5.7±1.2-fold increase, *p*<0.05), IL-8 (2.2±0.1-fold increase, *p*<0.01), and IL-10 (2.7±0.7-fold increase, *p*<0.05) (Fig. 5). Simultaneous exposure of the cells to NADPH and SPPs further augmented the production of IL-6, TNF-α, and IL-8; however, it induced statistically significant changes only in the case of IL-8 (1.7±0.5-fold increase, *p*<0.05, Fig. 5). Statistically insignificant changes in cytokine and chemokine levels were detected when using SPPs treated with DPI, SPP^H^, and SPP^H^ treated with DPI (Fig. 5). Taken together, these data indicate that the increased cytokine and chemokine production by SPP-treated moDCs can be considered as a response to oxidative stress generated by NAD(P)H oxidases present in SPPs.

### SPP-treated human moDCs stimulate allogeneic naïve T cell proliferation

**Figure 6:**
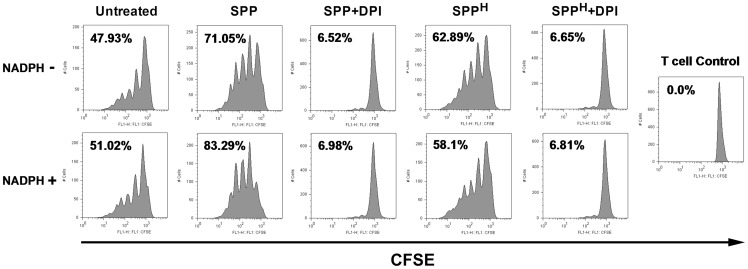
T cell-priming capacity of human moDCs exposed to SPPs. CFSE-labeled naïve CD4^+^ T cells were co-cultured with allogeneic moDCs pre-treated with SPPs under various conditions as indicated on the figure. After 5 days of co-cultivation, cell division was measured by flow cytometry. Numbers indicate the proportion of viable dividing T cells. Results are representative of four independent experiments. (DPI: diphenyleneiodonium; SPP: subpollen particle; SPP^H^: heat-inactivated SPP).

Activated DCs can trigger the development of adaptive immune responses; therefore, we studied the T cell-priming capacity of SPP-treated moDCs. Human moDCs were treated with SPPs under various conditions and then co-cultured with CFSE-labeled allogeneic naïve CD4^+^ T cells. The responsiveness of naïve CD4^+^ T lymphocytes to antigen presentation and cell viability were analyzed by flow cytometry after 5 days of stimulation. When treated with SPPs, moDCs induced proliferation in 71.1% of viable co-cultured T cells, while simultaneous exposure of moDCs to SPPs and NADPH led to a stronger induction of T lymphocyte proliferation (83.3%, Fig. 6). Pre-incubation of moDCs with DPI-treated SPPs, SPP^H^, and SPP^H^ treated with DPI decreased their T cell-priming capacity (6.5%, 62.9%, and 6.7%, respectively, Fig 6) and similar results were seen when these treatments were performed in the presence of NADPH (7.0%, 58.1%, and 6.5%, respectively; Fig 6). None of the treatments induced significant changes in cell viability (data not shown). These observations indicate that the T cell-priming capacity of SPP-treated moDCs depends, at least partly, on oxidative stress induced by SPPs' NAD(P)H oxidases.

### Effects of SPP treatment on the allostimulatory capacity of moDCs

**Figure 7:**
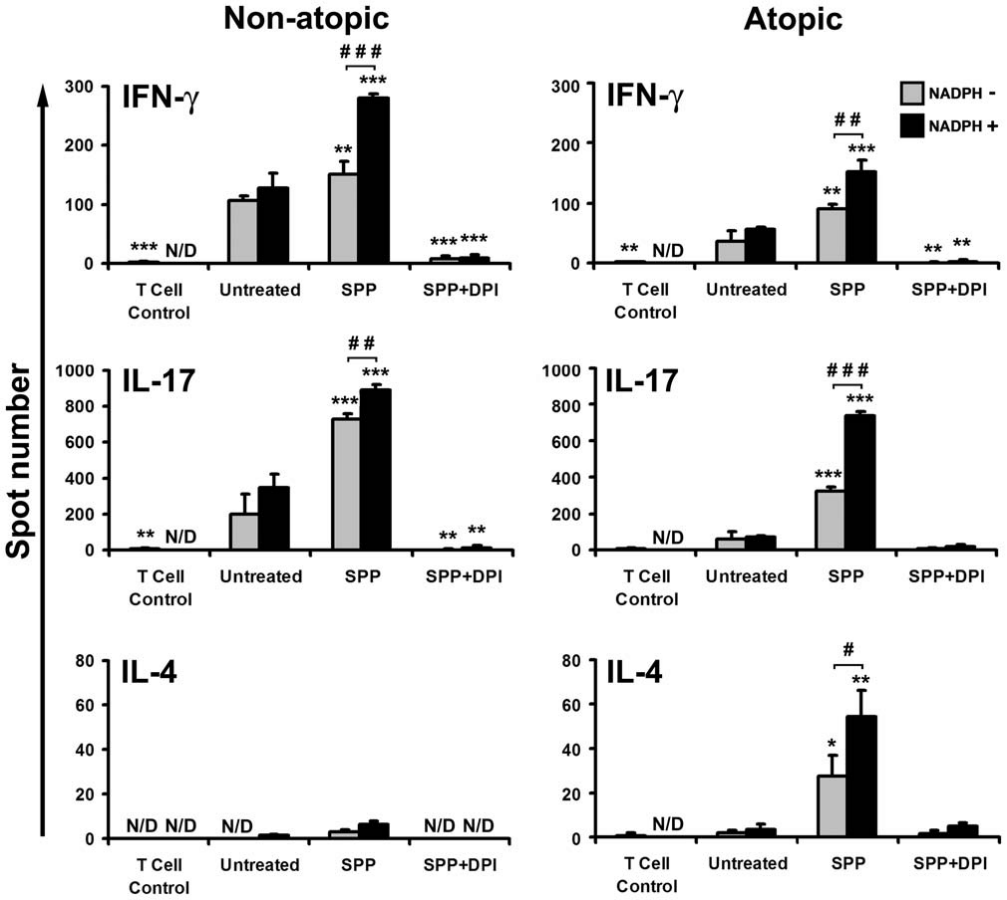
Cytokine production of CD3^+^ pan-T cells in response to co-culture with SPP-exposed human moDCs. Freshly isolated CD3^+^ pan-T cells obtained from three ragweed allergic individuals and three non-allergic ones were co-cultured with allogeneic moDCs pre-treated with SPPs for 4 days under various conditions as indicated on the figure. The numbers of IFN-γ-, IL-17-, and IL-4-producing T cells were detected by ELISPOT assays. Each assay was performed three times per donor, and data from one representative experiment are shown. Data are presented as means ± SEM. * *p*<0.05, ** *p*<0.01, *** *p*<0.001 vs untreated DCs, ^#^ *p*<0.05, ^##^ *p*<0.01, ^###^ *p*<0.001. (DPI: diphenyleneiodonium; N/D: not detectable; SPP: subpollen particle).

Human moDCs were treated with SPPs under various conditions and then co-cultured with freshly isolated CD3^+^ pan-T cells obtained from both ragweed-allergic and non-allergic individuals. Activation of CD3^+^ pan-T cells was assessed by IFN-γ, IL-17, and IL-4 ELISPOT assays. When SPP-treated moDCs were co-cultured with CD3^+^ pan-T cells from non-atopic individuals, a significant increase in the frequency of both IFN-γ- and IL-17-producing T cells was detected (*p*<0.01 and *p*<0.001, respectively) (Fig. 7). In parallel experiments, in which SPP-exposed moDCs were co-cultured with CD3^+^ pan-T cells from allergic donors, a significant rise not only in the frequency of IFN-γ- and IL-17-producing T cells but also in the frequency of IL-4-secreting T cells was observed (*p*<0.01, *p*<0.001 and *p*<0.05, respectively) (Fig. 7). These effects were significantly enhanced when moDCs were pre-incubated with SPPs in the presence of NADPH (Fig. 7). However, NADPH alone only slightly changed the capacity of moDCs to activate allogeneic T cells (Fig. 7). Due to a limited supply of T cells, we used, in this set of experiments only, DPI-treated SPPs as a control. Exposure to SPPs treated with DPI markedly reduced the allostimulatory capacity of moDCs in all experimental settings (Fig. 7). These findings suggest that the allostimulatory capacity of SPP-treated moDCs depends, at least partly, on oxidative stress induced by SPPs' NAD(P)H oxidases.

## Discussion

Previously, it was believed that conducting airway DCs have a rapid turnover due to continuous sampling of inhaled materials and migration to the mediastinal lymph nodes, even in the absence of infection or tissue damage in the lungs [31]. However, recent observations indicate that under steady-state conditions, only few mediastinal lymph node DCs actually originate from the airways and that inflammatory stimuli are generally necessary to provoke substantial accumulation of airway-derived DCs within lymph nodes [32]. Although intact ragweed pollen grains induce maturation and activation of DCs *in vitro* [24], [33], data from an *in vivo* study indicate that whole pollen grains only incidentally reach the broncho-alveolar part of the respiratory tract [34]. In this study we demonstrated that exposure to ragweed SPPs, which easily penetrate the lower airways and contain allergenic proteins and NAD(P)H oxidases, induces oxidative stress in human moDCs and triggers their activation program.

We recently found that moDCs possess efficient mechanisms to degrade exogenous ROS [26]. Despite this fact, SPP treatment induced a significant increase in intracellular ROS levels in moDCs. This is consistent with our previous data that SPPs increase ROS levels in epithelial cells [11]. In this study, we blocked SPPs' ROS producing ability with heat-treatment and/or DPI to reveal whether SPPs' NAD(P)H oxidases or internal oxidases are responsible for the observed oxidative stress in moDCs. Heat-treatment did not modify the size distributions of SPPs (Supl. Fig. S1), but notably reduced their capability to increase intracellular ROS levels indicating that other factors than simple uptake of particles contribute to oxidative stress in moDCs. The observation that DPI treatment nearly eliminates SPPs' ability to trigger oxidative stress in moDCs indicates that the increase in intracellular ROS levels is primarily due to SPPs' NAD(P)H oxidase activity. Indeed, exogenously added NADPH enhanced the ability of SPPs to elevate ROS levels in moDCs, implying either that exogenous NADPH gains entry to the SPPs by a yet undefined mechanism or that the NADPH binding site of pollen NAD(P)H oxidases is accessible for exogenous NADPH.

We also demonstrate that human moDCs internalize SPPs released from ragweed pollen, which is one of the most abundant aeroallergens. This is in line with previous observations that human alveolar macrophages phagocytose/bind allergen-containing SPPs from grass pollen [35]. Furthermore, a recent study has reported that in a complex three-dimensional human epithelial airway model, SPPs from timothy grass (*Phleum pratense*) were internalized by epithelial cells, monocyte-derived macrophages, and moDCs [36]. This uptake coincided with secretion of pro-inflammatory cytokines and chemokines. The authors concluded that an inflammatory effect of SPPs may also occur in the lung after a single inhalation of allergen particles and that may be independent of allergic sensitization to the particles [36]. These *in vitro* results suggest that pulmonary clearance of inhaled SPPs by professional phagocytes represents an essential primary defense mechanism *in vivo* to eliminate triggers of airway inflammation [37].

Our data showed that oxidative stress induced by exposure to SPPs upregulates both co-stimulatory and antigen presenting molecules on the surface of moDCs. This is consistent with a previous report that ROS generated by xanthine oxidase induce the phenotypic activation of DCs [38]. Furthermore, we recently established that intrinsic pollen NAD(P)H oxidases are responsible, at least partially, for enhancing the expression of activation and maturation markers on moDCs after contact with whole pollen grains [24]. It has been previously demonstrated that pollen-derived phytoprostanes [39] and adenosine [40] have regulatory potential on human DC function. In our experiments, the supernatant of SPPs did not modify the phenotype of moDCs, indicating that these soluble factors were removed from pollen suspension during isolation of SPPs by centrifugation. When SPP treatment was performed in the presence of NADPH, a substrate of pollen NAD(P)H oxidases, a further increase in the expression of all tested molecules was detected. Heat-inactivation, which eliminates the ROS-producing activity of SPPs [11], but does not affect the immunogenicity of most of the pollen proteins [41], did not completely abolish SPPs' ability to induce the up-regulation of CD40 and CD80. However, up-regulation of CD86 was significantly decreased on moDCs after exposure to SPP^H^ as compared to SPP treatment, suggesting a differential role of these co-stimulatory molecules in mediating pathogenesis of human allergic diseases similar to that reported in experimental murine models [42]. The findings with respect to relative fluorescence intensities from control experiments suggest that the uptake of DPI-treated SPPs decreases the expression of most markers below the basal levels. DPI, an inhibitor of flavoproteins, blocks the activity of plant NAD(P)H oxidases [43], human NADPH oxidase, and also mitochondrial NADH-ubiquinone oxidoreductase [44]. It has been shown that ROS are essential mediators in antigen presentation; a blockade of ROS generation by inhibitors of NADPH oxidase or by inhibitors of the mitochondrial electron transport chain significantly decreased T cell proliferation in response to antigen presentation by antigen presenting cells [45]. These previous findings and our observations indicate that during DPI treatment SPPs are loaded with the lipid soluble inhibitor [46] and the capture of these membrane-coated, DPI-containing particles by moDCs inhibits intracellular ROS generation, leading to a decreased expression of MHC class II and co-stimulatory molecules and abrogated T cell stimulatory capacity.

Several lines of evidence indicate that oxidative stress triggers transcriptional activation of pro-inflammatory cytokine and chemokine genes via nuclear factor κB and mitogen-activated protein kinase signaling pathways in macrophages and DCs [47]–[49]. However, oxidative stress also activates nuclear erythroid 2 p45-related factor 2 (Nrf2), a transcription factor that positively regulates many antioxidant genes [50]. A recent study demonstrated that DCs isolated from Nrf2-deficient mice secreted significantly higher amounts of IL-6 and TNF-α after exposure to ragweed pollen extract than DCs from wild-type mice [50]. Furthermore, we recently reported that ragweed pollen grain treatment increased the production of IL-8, TNF-α, and IL-6 by human moDCs, and this effect was decreased by antioxidants [24]. Here, we found that SPPs trigger the secretion of IL-6, TNF-α, IL-8, and IL-10 by moDCs through an oxidative stress-mediated mechanism. These findings underline the involvement of oxidative stress in pro- and anti-inflammatory mediator production by DCs after pollen or SPP exposure.

DCs are the most potent antigen-presenting cells; therefore, we also investigated the allostimulatory capacity of moDCs exposed to SPPs. We found that SPP treatment dramatically increases the ability of moDCs to prime T cells, and this depends, at least partly, on oxidative stress induced by SPPs' NADPH oxidases. Heat-inactivation of SPPs markedly reduced their ability to enhance the allostimulatory capacity of moDCs. These results are in agreement with our previous observations that ragweed pollen-treated moDCs strongly induced T lymphocyte proliferation and that exposure to heat-treated pollen grains or the presence of an antioxidant decreased the allostimulatory capacity of moDCs [24].

Previous studies show that allergen-specific T cells can be found in the blood of healthy individuals [51], [52]. In this study, when SPP-treated moDCs were co-cultured with CD3^+^ pan-T cells from atopic and non-atopic individuals, a significant increase in the frequency of both IFN-γ- and IL-17-producing T cells was detected as compared to control experiments. In contrast, an elevated frequency of IL-4-secreting T cells was observed only when SPP-exposed moDCs were co-cultured with CD3^+^ pan-T cells from allergic donors. These results confirm previous findings that stimulation with a house dust mite (HDM) allergen resulted in Th2 cytokine production by peripheral blood T cells from allergic but not from healthy children [52]. Furthermore, IFN-γ was induced in stimulated T cells from both healthy and HDM-allergic children [52]. Another study reported that T cells producing IL-17 in response to allergens exist in the peripheral blood of only sensitized atopic asthmatics [53]. A possible explanation for the difference between this finding and ours might be that the protease activity of HDM differently induces the expression of co-stimulatory molecules on the surface of DCs from healthy donors and pollen sensitive patients [54], while activation of DCs by ragweed pollen is not dependent on the immune status of the donors. Our observation that IL-4 production could be detected mainly when SPP-treated moDCs were co-cultured with T cells from ragweed allergic donors indicates that in our model exposure to SPPs induced the activation of ragweed-specific Th2 effector cells. Further studies are needed to reveal the frequency of ragweed-specific effector Th cells in the peripheral blood of atopic and non-atopic individuals.

It is widely accepted that at least two distinct signals are required to fully activate DCs [55]. The antigenic signal is derived principally through receptor-mediated antigen uptake, whereas another signal comes from the recognition of exogenous or endogenous danger. In this study, we demonstrated for the first time that SPPs released from ragweed pollen, which is one of the most abundant aeroallergens, can be uptaken leading to activation of human DCs and SPPs' NAD(P)H oxidase activity, which increases intracellular ROS levels, can enhance the activation stimuli. Based on our observations and the fact that they easily penetrate the lower respiratory tract, SPPs may have a decisive role in the sensitization phase of pollen-induced allergic reactions.

## Supporting Information

Figure S1 **Flow cytometric and microscopic visualization of the freshly isolated SPPs and heat-inactivated SPPs.** Freshly isolated SPPs and heat-inactivated SPPs were fluorescently labeled with CellVue® Jade Dye (45 min, 4°C; Polysciences Inc., Warrington, PA). To eliminate excess probe, SPPs were transferred to a separate vial, washed twice, pelleted by centrifugation (9000 g, 15 min, 4°C), and resuspended in PBS. (A) Flow cytometric analysis was used to characterize the Forward Scatter parameters (FSC-H) of the SPPs samples that correlate with particle size. (B) For laser scanning confocal microscopy, fluorescently labeled SPP samples were mounted on microscopic slides with Mowiol 4–88 (Calbiochem, Darmstadt, Germany) under the coverslip to reduce unwanted photobleaching. The CellVue® Jade-labeled SPPs were excited at 488 nm and fluorescence emission was detected through 505 to 550 nm filters using a Zeiss LSM 510 microscope (Carl Zeiss AG, Jena, Germany) with 40× C-Apochromat water immersion objective (NA1.2). Scale bar  = 2 µm (upper) and 1 µm (lower).
(TIF)
