# Supplementary figures and images for: Ragweed Subpollen Particles of Respirable Size Activate Human Dendritic Cells
Source: PLoS One. 2012 Dec 14;7(12):e52085. doi: 10.1371/journal.pone.0052085 (PMC3522620; doi:10.1371/journal.pone.0052085)

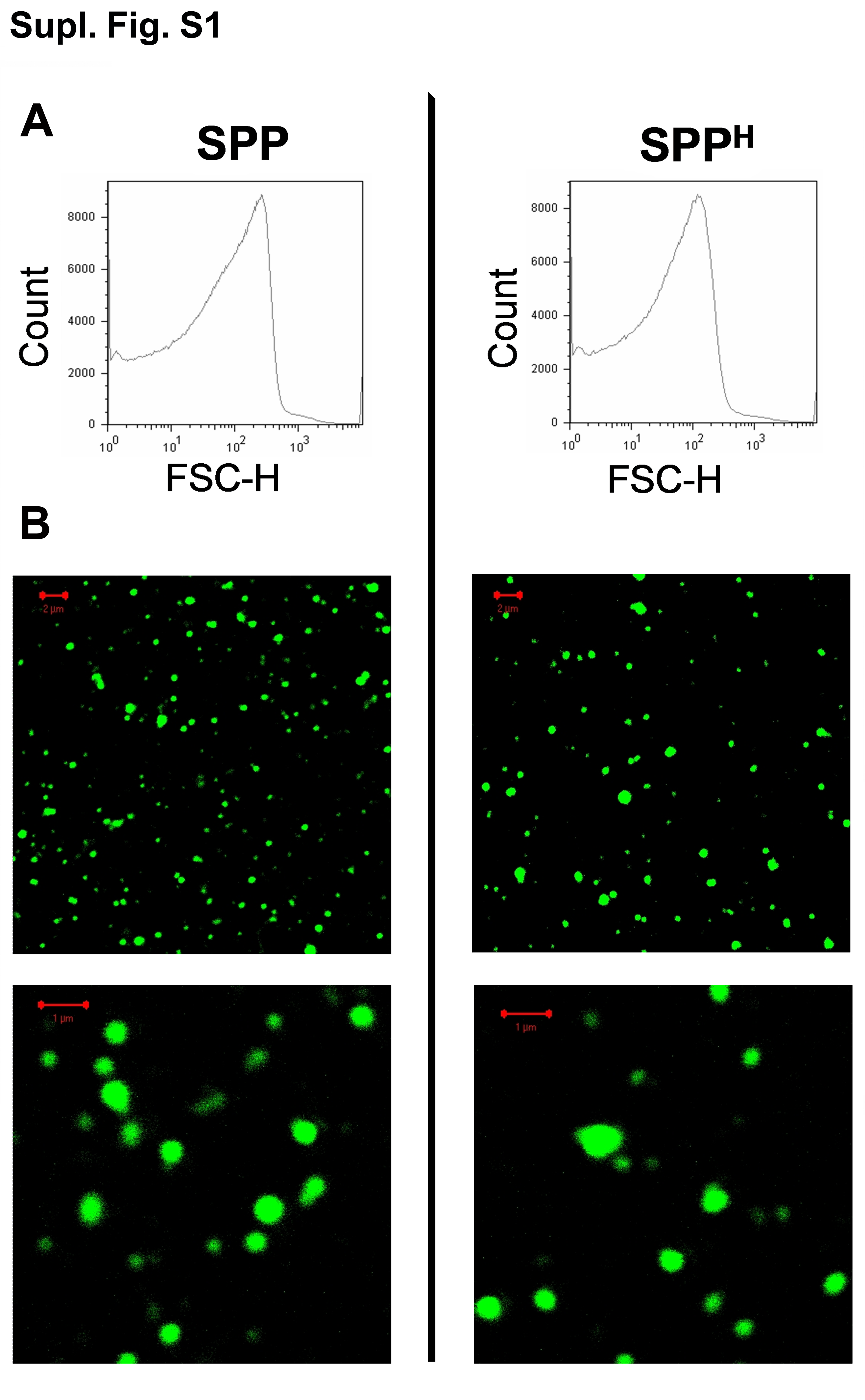

Supplement: Figure S1 — Flow cytometric and microscopic visualization of the freshly isolated SPPs and heat-inactivated SPPs. Freshly isolated SPPs and heat-inactivated SPPs were fluorescently labeled with CellVue® Jade Dye (45 min, 4°C; Polysciences Inc., Warrington, PA). To eliminate excess probe, SPPs were transferred to a separate vial, washed twice, pelleted by centrifugation (9000 g, 15 min, 4°C), and resuspended in PBS. (A) Flow cytometric analysis was used to characterize the Forward Scatter parameters (FSC-H) of the SPPs samples that correlate with particle size. (B) For laser scanning confocal microscopy, fluorescently labeled SPP samples were mounted on microscopic slides with Mowiol 4–88 (Calbiochem, Darmstadt, Germany) under the coverslip to reduce unwanted photobleaching. The CellVue® Jade-labeled SPPs were excited at 488 nm and fluorescence emission was detected through 505 to 550 nm filters using a Zeiss LSM 510 microscope (Carl Zeiss AG, Jena, Germany) with 40× C-Apochromat water immersion objective (NA1.2). Scale bar = 2 µm (upper) and 1 µm (lower). (TIF) [file pone.0052085.s001.tif]
